# Effects of Age and Social Adversity on Immune Cell Populations in a Non-Human Primate Model of Human Aging

**DOI:** 10.1093/geroni/igab046.2044

**Published:** 2021-12-17

**Authors:** Mitchell Sanchez-Rosado, Noah Snyder-Mackler, James Higham, Lauren Brent, Nicole Marzan-Rivera, Melissa Pavez-Fox, Marina Watowich, Carlos A Sariol

**Affiliations:** 1 University of Puerto Rico Medical Sciences Campus, San Juan, Puerto Rico, United States; 2 Arizona State University, Tempe, Arizona, United States; 3 NYU, New York University, New York, United States; 4 University of Exeter, Exeter, England, United Kingdom; 5 UPR-Medical Sciences Campus, UPR-Medical Sciences Campus, Puerto Rico, United States; 6 University of Washington, Tempe, Arizona, United States

## Abstract

Significant hallmarks of aging are immune function decline and rising cumulative inflammation. These immunosenescent signatures are also found in individuals who experience chronic social adversity, independently of age. However, no studies to date have examined how social adversity alters immune function across the lifespan –data that are essential to identify the molecular routes through which social adversity might lead to increased aging-related disease. Over a two-year period, we investigated how age and social adversity (quantified by low social status) affected immunity. We measured immune cell proportions at baseline and their gene regulation after in vitro stimulation with pathogen molecules that stimulated both Th1 and Th2 immune responses in a population of free-ranging rhesus macaques. We first performed flow cytometry on peripheral whole blood to quantify changes on immune cell proportions across the lifespan (n=235) and in animals of different social statuses (n=141). We found significant decreases in CD20+ B cells and CD3+/CD4+ T cell proportions with age, suggesting diminished antibody production and adaptive immune responses in older individuals. Age-associated increases in CD3+/CD8+, CD3+/CD4+/CD25+ T regulatory cells and CD14-/CD16+/HLA-DR+ non-classical monocytes indicated heightened baseline inflammation in older animals. Social adversity recapitulated the effects of aging in CD14+/CD16-/HLA-DR+ classical monocytes, indicating immune deficits in phagocytosis and pathogen clearance in older and lower status individuals. Using RNA-seq, our stimulations (n=1,320) will allow us to identify molecular immune pathways that are disrupted by age and social adversity, similarities in response between age and adversity, and how the effect of adversity varies across the lifespan.

